# Acral vascular necrosis associated with immune-check point inhibitors: case report with literature review

**DOI:** 10.1186/s12885-019-5661-x

**Published:** 2019-05-14

**Authors:** Karam Khaddour, Veerpal Singh, Maryna Shayuk

**Affiliations:** 1 Rosalind Franklin University of Medicine and Science, 915 Armistead Lane, McHenry, Chicago, IL 60050 USA; 2 North Western Medicine Centegra Healthcare System, Chicago, USA

**Keywords:** Acral ischemia, Necrosis, Case reports, Small vessel vasculitis, Immunotherapy, Immune related adverse events (IRAEs)

## Abstract

**Background:**

Treatment of solid malignancies has been revolutionized with the introduction of immune checkpoint inhibitors (ICIs) and their use is being expanded in therapy of different cancers. However, immune related adverse events (IRAEs) can occur during treatment. These side effects occur due to stimulation of the innate and adaptive immune system and can lead to serious complications. Recently, acral ischemia has been reported in some cases during treatment with programmed death-1 (PD-1) and cytotoxic T lymphocyte associated antigen-4 (CTLA-4) inhibitors. Here, we discuss a case in which acral necrosis developed after initiation of a PD-1 inhibitor. We offer a review of the existing literature on the pathophysiology, clinical course and treatment outcomes.

**Case presentation:**

A 68-year-old female was diagnosed with stage IV non-small cell lung adenocarcinoma and was started on pembrolizumab. The patient developed sudden onset numbness and discoloration of fingertips bilaterally at week 25 after initiation of ICI treatment. Extensive workup to rule out hypercoagulable, autoimmune and vascular disease was unremarkable except for mild elevation of ANA and ESR. The symptoms quickly progressed into dry gangrene within four weeks and did not respond to medical or surgical treatment. Pembrolizumab was subsequently discontinued due to progression of metastatic disease. The patient refused further interventions and transitioned to hospice care where she expired after two months.

**Conclusion:**

Acral ischemia can develop during treatment of malignancies. This complication, although uncommon, canresult in digital amputation. Physicians should be aware of the possible progression of acral vascular necrosis when Raynaud’s like symptoms develop. Larger studies are needed to confirm the role of ICIs in the pathogenesis of acral vascular necrosis.

## Background

In May 2017, BMC Cancer Journal published a case report by Gambichler et al. describing development of paraneoplastic acral vascular necrosis in association with CTLA-4 and PD-1 inhibitors during treatment of metastatic melanoma [1]. Other reports described similar findings under different diagnostic terms including small vessel vasculitis and digital ischemia. This newly observed adverse event although not reported in phase I/II trials appears to be significant should necrosis progresses requiring surgical amputation. Immune checkpoint inhibitors (ICIs) enhance the immune system through blockage of costimulatory signal receptors that are present on normal and cancer cells, which facilitate tumor evasion by inducing tolerance and anergy. The resultant adverse events of hyperstimulation of the immune system during treatment with ICIs are termed immune related adverse events (IRAEs). These side effects can have a wide spectrum of manifestations such as acute hypophysitits, colitis, pneumonitis and rarely myocarditis. Moreover, vasculitis has been described to occur during treatment mostly in large and medium size vessels. Recently, some reports suggested an association between ICIs and small vessel vasculitis, which might lead to digital ischemia and necrosis.

## Case presentation

**Fig. 1:**
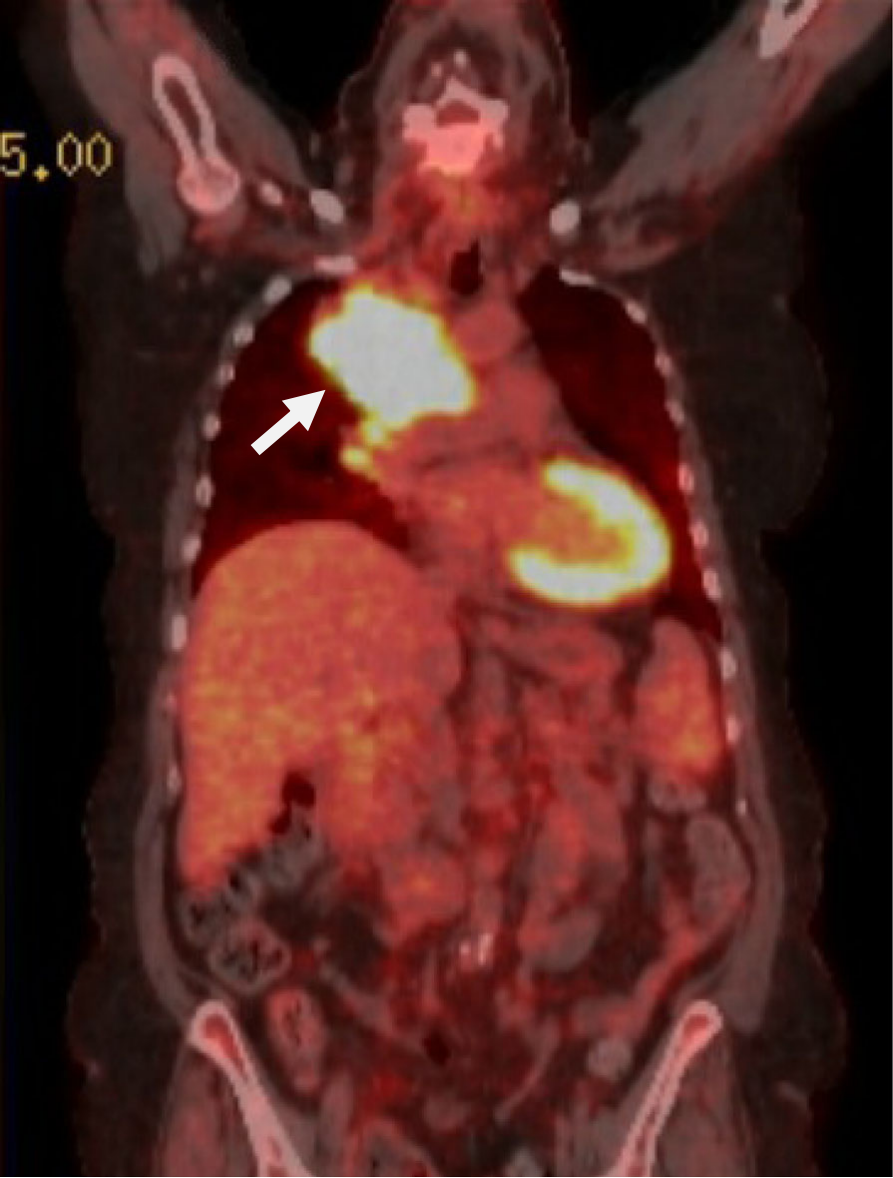
Coronal PET-CT Demonstrating the Primary Lung Tumor. Legends: Coronal PET-CT indicating right upper lobular mass (white arrow) measuring 8.5 × 5.5 in the biaxial diameter

**Fig. 2:**
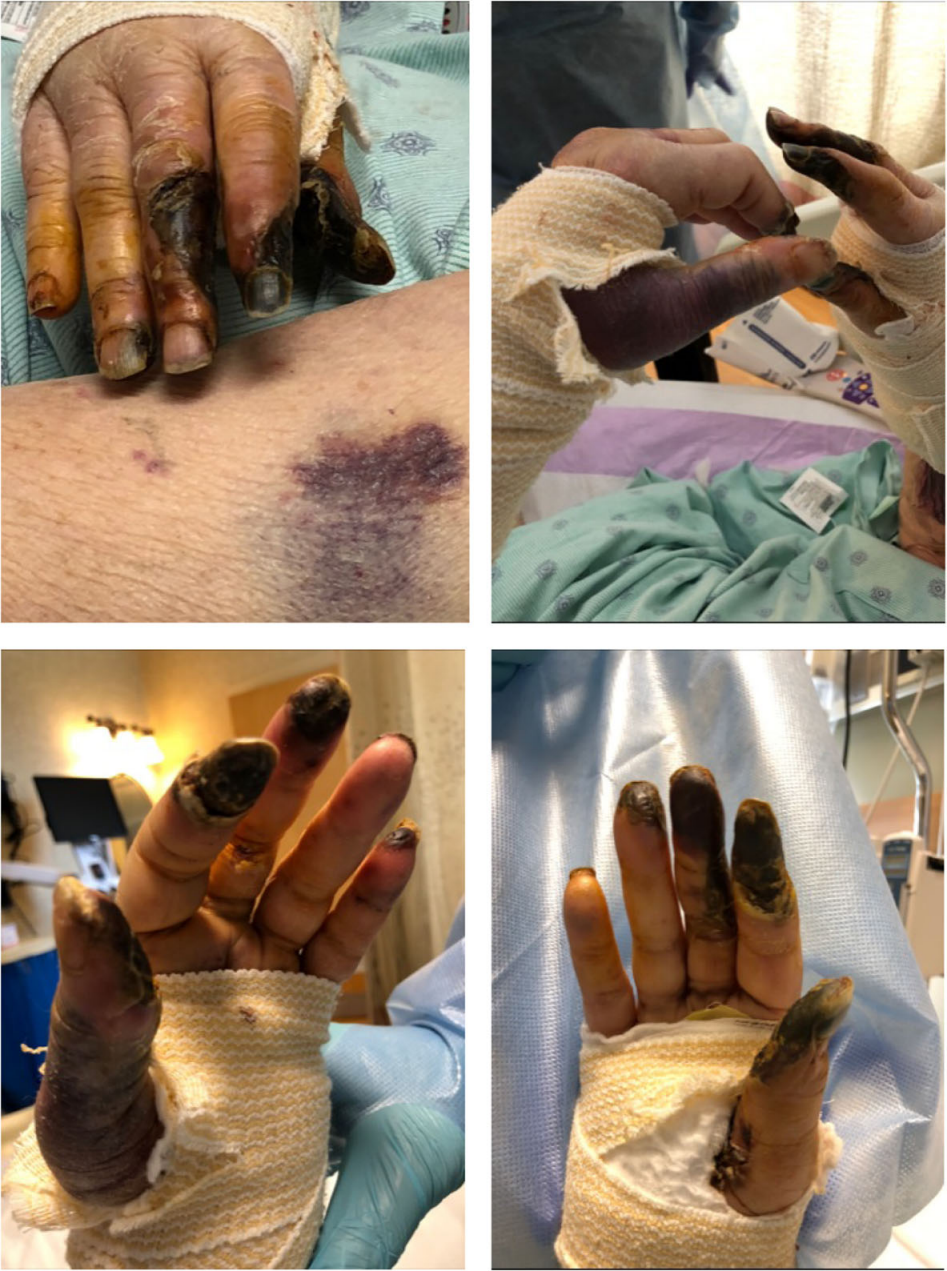
Development of acral necrosis at week 33 of treatment with PD-1 inhibitors. Legends: Varying degree of extension of necrosis and ulceration involving the proximal phalanges in right (1st^,^ 2nd^,^ 3rd^,^ 4th) ^and^ (1st^,^ 2nd^,^ 3rd^,^ 5th) digits in the left hand. There is no presence of calcinosis or involvement of the bone protuberants

A 68-year-old Caucasian female presented to our hospital with shortness of breath and unintentional weight loss of 30 pounds three months prior. The patient was an active smoker and her past medical history included well controlled type 2 diabetes mellitus (Hemoglobin A1C 6.0%) and non-obstructive coronary artery disease. Her medication included insulin, aspirin and metoprolol succinate. Thoracic computed tomography showed an interlobular mass in the medial right upper lobe with extension into the right hilum (Fig. 1). Tissue biopsy was performed and histopathology was consistent with non-small cell adenocarcinoma with negative EGFR mutation, ALK and ROS-1. Programmed Death Ligand-1 (PD-L1) expression by immunohistochemistry was 70%. The clinical staging with Positron Emission Tomography- Computed Tomography (PET-CT) scan showed mediastinal metastatic lymph nodes and scattered osseous metastases in the axial and proximal appendicular skeleton (Stage IV; T4, N3, M1b). Given the high expression of PD-L1 > 50%, immunotherapy with pembrolizumab was started at (200 mg) intravenously once every three weeks per KEYNOTE-024 protocol [2]. Subsequent surveillance with CT showed a significant decrease in the size of the primary tumor in the lungs from 8.5 × 5.5 cm to 2.7 × 0.9 cm with a decrease in the size of bone metastases. In addition, patient’s Eastern Cooperative Oncology Group performance status (ECOG) improved from 2 prior to therapy to 0. After week 25 of pembrolizumab, she developed Raynaud’s like symptoms in both hands with mild non-purpuric erythema, pain and paresthesia at the fingertips bilaterally aggravated by cold weather. A thorough history revealed no prior autoimmune disease, recent trauma or similar symptoms in the past. The patient was started on nifedipine 30 mg extended release orally once daily due to potential vasospasm demonstrated by the triad of pallor, cyanosis and hyperemia. There was no improvement of symptoms on 1 week follow up and nifedipine dose could not be increased due to labile blood pressure. The mild intermittent discoloration progressed to persistent periungual blue discoloration over the course of 2 weeks in the digits bilaterally. The patient was started on oral prednisone 30 mg daily (0.5 mg per kg daily) in conjunction with nifedipine. An autoimmune panel revealed anti-nuclear antibodies (ANA) of 1:80 with a homogenous pattern; erythrocyte sedimentation rate (ESR) of 70 mm/hr. Further testing, including ANCA-c, ANCA-p was negative. Complement levels were within normal limits in our lab reference range. Simple nailfold capillaroscopy did not yield any abnormalities. Additional work up did not reveal a septic source, hypercoagulable disease, hyperviscosity related immunoglobulinopathies, lymphoproliferative disorders or vascular occlusion as shown in Table 1. The patient continued on pembrolizumab given the significant interval tumor response. Over the course of 2 weeks, the patient experienced progression of the discoloration into dry gangrene, ulceration with necrosis involving all fingertips and extending to the proximal phalanges in right digits (1st^,^ 2nd^,^ 3rd^,^ 4th) and (1st^,^ 2nd^,^ 3rd^,^ 5th) digits in the left (Fig. 2). Arterial Doppler ultrasound showed normal arterial waveforms through the upper extremity arteries with peak systolic velocity ratios within normal limits. There were no signs of stenosis, atherosclerotic disease or occlusion. CT angiography showed a normal appearance of the arterial branch up to the palmar arch artery without evidence of saccular aneurysm formation, stenosis or occlusion; digital arteries were not adequately visualized. The patient underwent sympathectomy given the rapid progression of necrosis with no response to calcium channel blockers or glucocorticoids. The decision of amputation was deferred until necrotic tissue would have clear demarcation (Table 2 shows course of progression).

**Table 1:** Laboratory and imaging tests performed after development of acral ischemia

	Tests Performed	Results
Infection	CBC, CRP Blood cultures Echocardiography HBV, HCV	Normal Negative No vegetation or myxoma Negative
Hypercoagulable state	Protein C Protein S Anti- thrombin 3 Platelet PT/aPTT/INT	131% (Normal) 73% (Normal) Normal 280 10 × 3/uL (Normal) Normal
Lymphoproliferative disease	Kappa/Lambda Ratio Beta-2 microglobulin (B2M) LDH SPEP/IFE	1.37 (Normal) 3 mg/L (Mildly elevated) 138 U/L (Normal) Normal
Tumor invasion	CT Chest CT Angiography	No invasion of sympathetic nervous plexus No vascular occlusion, saccular aneurysm or stenosis up to the palmar arch arteries
Autoimmune and inflammatory disease / Thromboangiitis obliterans	ANA ESR CH50 ANCA-c, ANCA-p Cryoglobulin APL ab, Anti-Scl-70 Ab, Anti-dsDNA ab, Anti-U1 RNP Ab, Anti-Sm ab, Anti-Ro/SSA ab Nailfold videocapillaroscopy Skin biopsy	**1:80 Homogeneous** **70 mm/hr** 170 CAE Units (elevated) (Acute phase reactant) Normal Negative (Normal) Not performed Not performed Not performed

*Ab: antibodies, APL: antiphospholipid, Anti-Scl-70: topoisomerase I, ANCA-c: Central anti-neutrophil cytoplasmic antibodies, ANCA-p: perinuclear anti- neutrophil cytoplasmic antibodies, Anti-Sm: anti smith antibodies, Anti-Ro/SSA: anti sj*ö*gren’s syndrome related antigen A, SPEP: serum protein electrophoresis, IFE: immunofixation electrophoresis*

**Table 2:** Timeline of progression of acral necrosis since administration of PD-1 inhibitor

Timeline from Treatment	Clinical Progression
Day 0	1st dose of pembrolizumab (No symptoms)
At 25 weeks	Raynaud’s like symptoms (Patient started on nifedipine)
At 27 weeks	No resolution of Raynaud’s symptoms and persistence of cyanosis (Patient started on prednisone)
At 29 weeks	Development of dry gangrene at fingertips bilaterally
At 33 weeks	Extension of necrosis and development of ulceration. Patient still on pembrolizumab. Sympathectomy performed (patient still on nifedipine and glucocorticoids)
At 37 weeks	Tumor progression (pembrolizumab stopped). Extension of the necrosis stopped after week 37

Pembrolizumab was stopped later (week 37) due to tumor progression and it was noted that the involved necrotic area in the digits did not progress further after discontinuation of PD-1 inhibitor. Unfortunately, the patient transferred to hospice care and expired two months after.

## Discussion and conclusion

Acral necrosis has not been documented to occur as an immune related adverse event (IRAEs) during phase I/II trials of CTLA-4 and PD-1 inhibitors [3, 4]. However, a recent French prospective multicenter study (REISAMIC) was conducted to assess the development of connective tissue disease after treatment with PD-1/PD-L1 inhibitors [5]. This study found the incidence to be low with only three of 447 patients who developed connective tissue disease. Interestingly, one of the three patients had small vessel vasculitis involving the digits, which was secondary to cryoglobulinemia and was associated with an elevated ANA (1:160) and anti-SSA antibodies [5]. The most common type of vasculitis that develops as an (IRAEs) involves large and medium vessels as was demonstrated in a case series by Daxini et al. [6]. This systematic review reported 20 cases of vasculitis associated with treatment with ICIs of which three were small vessel vasculitis one of them being digital ischemia [6]. There have been more cases reported in the last two years describing progression of acral necrosis, which might represent small vessel vasculitis during treatment with ICIs.

The time onset of acral ischemia developing after initiation of ICI treatment varies in the different cases reported between 3 and 26 weeks as noted in Table 3. Similar results of previous case series have reported duration of 1.2 to 6 months before development of vasculitis after initiating treatment with ICIs [6]. Vascular risk factors such as diabetes and smoking have been reported in some patients who developed digital ischemia during treatment with ICIs. None of the patients reported in the literature had a preexisting autoimmune disease prior to initiating immunotherapy treatment. A comprehensive work up is recommended to identify the etiology of the acral ischemia which can dictate treatment strategy (Table 1 illustrates some of the tests that could help establishing a diagnosis). History and physical exam remain essential as they can guide with diagnostic work up for acral necrosis, which should consider all possible etiologies. Optimal treatment of acral necrosis in association with ICIs remains unknown given the low number of observations and multidisciplinary approach is warranted. The use of calcium channel blockers, prostaglandins and sympathectomy reported in different cases have all been unsuccessful [1, 7]. Glucocorticoids showed variable outcomes from complete resolution of the ischemia to partial or no response [1, 8]. Rituximab was reported to be beneficial in one case and to halt the progression of acral necrosis, but the patient required digital amputation eventually [7].

**Table 3:** Summary of case reports of digital ischemia and small vessel vasculitis with immune-check point inhibitors use

Case	Age /Gender	Vascular Risk Factors	Malignancy	Medication	Symptoms	Duration before onset of symptoms	Treatment	Final Outcome
Gambichler et al. [1]	60/ Male	None	Metastatic Melanoma BRAF V600E Mutated	Ipilimumab + Nivolumab	Subungal necrosis followed by gangrene	3 weeks	Prostacycline, methylprednisolone (50 mg in tapering dose), methylprednisolone 500 mg	Surgical amputation
Padda et al. [7]	52/ Female	None	Metastatic Melanoma	Ipilimumab	Digital Necrosis	3 weeks	Amlodipine, Aspirin, Prednisone (60 mg), methyl prednisolone 500 mg, epoprostenol, Normal Saline, Rituximab	Surgical amputation
Thoreau et al. [12]	73/ Male	Diabetes	Metastatic Melanoma	Pembrolizumab	Acute ischemia of the left toes	26 weeks	Iloprost, anticoagulation, amputation, aspirin, fogarty arterial embolectomy	Surgical amputation
Comont et al. [8]	66/ Male	Smoker	Urothelial Bladder Cancer	Tremelimumab + durvalumab/Chemotherapy^a^	Periungal skin necrosis bilaterally	20 weeks	Prednisone 1 mg/kg Immunotherapy discontinuation	Complete resolution
Leburel et al. [5]	60/Male	Not available	Melanoma	PD-L1 inhibitor+ BRAF and MEK inhibitors	Cyanosis of fingers, necrosis of 3 fingers and the heels, arthralgia, dry mouth, paresthesia of feet and interstitial pneumonia	8.5 weeks	ICI withdrawal, prednisone 1 mg/kg, CCB, iloprost and ASA	Partial resolution

^a^Methotrexate, vinblastine, doxorubicin and cisplatin

*ASA* acetylsalicylic acid, *CCB* calcium channel blockers

We postulate two hypotheses to explain the pathophysiology for development of acral necrosis during treatment with ICIs. The first hypothesis is based on the anecdotal reports and the mechanism of action of ICIs that leads to alteration of the immunological homeostasis. This could lead to either activation of T cell population or antibody formation against self-antigens (endothelial cells in this case) which theoretically could cause vasculitis related syndromes. Zhang et al. examined the role of PD-1/PD-L1 inhibition on the development of vasculitis specifically giant cell arteritis and concluded that blockade of the coninhibitory ligand can initiate T cell infiltration of the vascular endothelium and exacerbate an inflammatory response that leads to vasculitis [9]. However, Zhang’s study involved giant cell arteritis, which is a medium/ large vessel vasculitis. Moreover, PD-1 receptor impairment has been described to induce autoantibodies against shared antigens between the tumor and normal tissue knock-out mice models leading to lupus-like syndrome [10]. Some literature regarding digital ischemia favors autoimmune involvement during treatment with ICIs. As an example, Comont et al. described a case of acral necrosis during combined treatment with CTLA-4 and PD-L1 inhibitors that was associated with increased titers of ANA (1:5200) which would support an autoimmune etiology [8]. In this case, there was a complete reversal of the acral ischemia with high suppressive dose of prednisone (1 mg/kg daily) [8]. However, the patient of the previous study received chemotherapy prior to ICI includeding methotrexate, vinblastine, doxorubicin and cisplatin which could be culprits in acral necrosis. Similarly, a patient who developed digital ischemia in (REISAMIC) study had high ANA titers (160, speckled pattern) and responded well to steroids with partial resolution of ischemic symptoms [5]. In our patient, there was a weak evidence of an autoimmune process due to borderline ANA and elevated ESR which were nonspecific for a definitive diagnosis for autoimmune conditions as they can be elevated in various non-immunologic conditions and our patient did not have a good response to prednisone (received prednisone0.5 mg/kg/day). In addition, Gambichler et al. performed a tissue biopsy from the area of acral necrosis in their patient, which did not reveal any evidence of T cell infiltration or immune complex precipitation that might represent leuococytoclastic vasculitis [1].

The second hypothesis for the development of acral necrosis with ICIs treatment is the proinflammatory effect causing vascular damage. The endothelial insult could induce either atherosclerotic lesions or a procoagulable state, which might lead to vascular (arterial) thrombosis. Mice models that lacked PD-1 receptors due to PD-1 blockade had more abundant T cell inflammatory infiltrate in atherosclerotic lesions compared to control mice models suggesting that PD-1 impairment can lead to proatherogenic state [11]. In our search of the literature there was one case that involved acral ischemia of left toes with the use of PD-1 inhibitors. The patient was later found to have arterial thrombosis, which was removed with fogarty thrombectomy and was believed to be secondary to PD-1 inhibitor. However, the patient had diabetes mellitus with diabetic ketoacidosis, which also could induce endothelial inflammatory damage under oxidative stress leading to arterial thrombosis [12].

Historically, the observed phenomenon of acral ischemia was described early in the literature in association with malignancy and was considered a manifestation of a paraneoplastic syndrome [13]. The pathophysiology of paraneoplastic acral vascular syndrome (PAVS) is not well established. Many cases described development of acral ischemia with specific chemotherapies including gemcitabine and carboplatin leading to think that medication could be a culprit in the development of acral ischemia [14, 15]. Older generation immunotherapies like interferon may potentially cause acral ischemia as noted in a case series by Sharpai et al. which identified Raynaud’s like syndrome and acral arterial occlusion in 12 patients of 24 who were treated with interferon [16]. Other studies have emphasized these findings and have showed interferon alpha/beta to cause endothelial injury in the microvasculature leading to impaired subcutaneous tumor blood flow in mice models [17]. Paraneoplastic acral vascular necrosis is more frequently encountered in association with adenocarcinoma followed by squamous cell carcinoma and hematologic malignancies from a cohort of 100 patients who were hospitalized due to digital ischemia [18]. This study found an incidence of 15% of malignancy associated with digital ischemia [18].

To our knowledge, our report is the first to describe development of acral necrosis with the use of pembrolizumab, which is a humanized monoclonal antibody against programmed death 1 receptors [19].

Acral vascular necrosis can develop in association with malignancies and during treatment. It is controversial whether this complication represents an immune related adverse event to the use of ICIs. Physicians should have a high index of suspicion when Raynaud’s like symptoms occur during treatment of malignancy and monitor closely for development of digital ischemia and necrosis. Larger studies are needed to identify the etiology of this complication.

## Abbreviations

ANA: Antinuclear antibodies
CTLA-4: Cytotoxic T lymphocyte antigen-4
ICIs: Immune-check point inhibitors
IRAEs: Immune related adverse events
PAVS: Paraneoplastic acral vascular syndrome
PD-1/PD-L1: Programmed death-1/ programmed death- ligand 1
